# Hypoxia-Driven Immunosuppressive Metabolites in the Tumor Microenvironment: New Approaches for Combinational Immunotherapy

**DOI:** 10.3389/fimmu.2018.01591

**Published:** 2018-07-16

**Authors:** Yiliang Li, Sapna Pradyuman Patel, Jason Roszik, Yong Qin

**Affiliations:** ^1^Key Laboratory of Radiation Medicine and Molecular Nuclear Medicine, Institute of Radiation Medicine, Peking Union Medical College & Chinese Academy of Medical Sciences, Tianjin, China; ^2^Department of Melanoma Medical Oncology, The University of Texas MD Anderson Cancer Center, Houston, TX, United States

**Keywords:** hypoxia, immunosuppression, metabolism, immunotherapy, microenvironment

## Abstract

Hypoxia is not only a prominent contributor to the heterogeneity of solid tumors but also a crucial stressor in the microenvironment to drive adaptations for tumors to evade immunosurveillance. Herein, we discuss the potential role of hypoxia within the microenvironment contributing to immune resistance and immune suppression of tumor cells. We outline recent discoveries of hypoxia-driven adaptive mechanisms that diminish immune cell response *via* skewing the expression of important immune checkpoint molecules (e.g., cluster of differentiation 47, programmed death ligand 1, and human leukocyte antigen G), altered metabolism and metabolites, and pH regulation. Importantly, inhibition of hypoxic stress-relevant pathways can collectively enhance T-cell-mediated tumor cell killing. Furthermore, we discuss how manipulation of hypoxia stress may pose a promising new strategy for a combinational therapeutic intervention to enhance immunotherapy of solid tumors.

## Introduction

To survive and grow, tumors insulate themselves with various layers of immunosuppressive stroma to locally disable and/or evade the immune system. It is known that the tumor microenvironment contributes to the heterogeneity of the tumor and supports tumor growth and resistance to systemic therapies (1). In solid tumors, a hypoxic area is a common structural characteristic and some tumor cells exist in a hypoxic environment, whereas some exist in a vascularized area with sufficient oxygen supply (2). Numerous studies have shown that hypoxia is strongly associated with lower overall survival and disease-free survival of various tumor types (3–5). Notably, hypoxic tumor cells are considered to be more aggressive and more resistant to conventional systemic therapies and radiotherapy than non-hypoxic tumors (6, 7). Besides increasing the expression of various genes involved in angiogenesis and drug-resistance, hypoxia also promotes the selection of apoptosis-resistant clones and induces metastasis (8). Although the roles of hypoxic stress in the crosstalk among immune cells, stroma components, and tumor cells are not fully elucidated, it is widely appreciated that the hypoxic zone in solid tumors induces immune tolerance by impeding the homing of immune effector cells into tumors. Several regulatory mechanisms related to redundant levels of immune suppression and functional heterogeneity driven by hypoxia in the tumor microenvironment have been identified (9, 10).

## Hypoxic Regulation of the Expressions of Immunosuppressive Molecules in the Tumor

**Figure 1:**
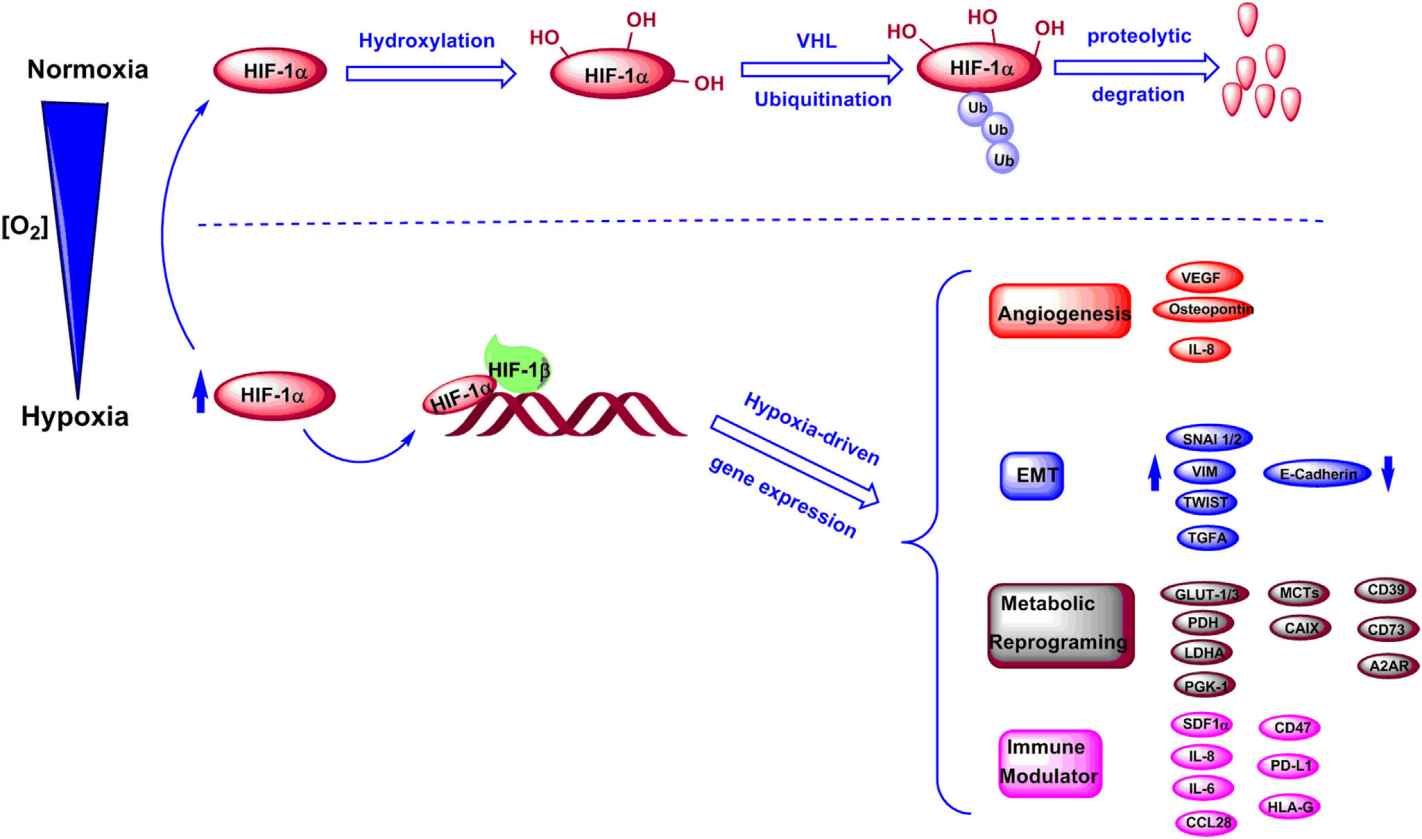
Regulation of HIF-1α levels and downstream genes under normoxic and hypoxic conditions. Under normoxic conditions, HIF-1α is hydroxylated and further undergoes degradation through an ubiquitination-dependent process mediated by VHL. Under hypoxic conditions, HIF-1α is stabilized and forms a complex with HIF-1β, which induces transcriptions of various genes involved in angiogenesis, epithelial–mesenchymal transition (EMT), metabolic reprogramming, and immune regulation.

The key mediators of hypoxic signaling are hypoxia-inducible factors (HIFs). HIFs are a family of transcription factors consisting of three alpha subunits, HIF-1α, HIF-2α, and HIF-3α that can heterodimerize with HIF-1β (11). HIF transcriptional activity is known to be oxygen-dependent. Under normoxic conditions, conserved proline residues on HIF-1α are hydroxylated by proly-4-hydroxylase (PHD), and the hydroxylated HIF-1α can be downregulated by ubiquitination and proteasomal degradation mediated by von Hippel−Lindau protein (11, 12) (Figure 1). Under hypoxic stress, induction and stabilization of HIF-1α and/or HIF-2α lead to upregulation of transcription of numerous hypoxia-responsive genes related to metabolic and immune pathways, and resulting in modulation of both metabolism and immunity of tumor and stromal cells (12, 13) (Figure 1). Particularly, hypoxia has been proven to contribute to increased angiogenesis through upregulation of interleukin 8 (IL-8), osteopontin, and vascular endothelial growth factor (13–17). Notably, hypoxic tumor microenvironment was correlated to a high expression of genes that promote epithelial–mesenchymal transition (EMT), including inhibitor of differentiation 2, snail 1 and 2 (*SNAI1* and *SNAI2*), transcription factor 3, transforming growth factor alpha, twist transcription factor (*TWIST*), vimentin (*VIM*), and zinc finger E-box-binding homeobox 1 and 2 (*ZEB1* and *ZEB2*) (18–22). Moreover, hypoxia has been shown to downregulate E-cadherin expression in tumors affecting cell–cell adhesion (22, 23). Recent evidence shows that the hypoxia-HIF-EMT niche is considered to support maintenance of cancer stem cells (24, 25). Hypoxic regions in tumors can lead to long-lasting HIF signaling, which is known to function as an oncogenic stimulus in some settings driving cancer development, invasion, immune suppression, and metastasis (25–27) (Figure 1).

In addition to tumor cells and stromal cells, the hypoxic regions in solid tumors have been found to be infiltrated by high levels of immunosuppressive cells, such as myeloid-derived suppressor cells (MDSCs), tumor-associated macrophages (TAMs), and T-regulatory (Treg) cells (10, 28). The mechanisms of recruitment and function of these immunosuppressive cells within the hypoxic tumor microenvironment have also been studied (29). Hypoxia has been shown to induce the production of stromal cell-derived factor 1α (SDF1α) by hypoxic tumor cells, which binds to C-X-C chemokine receptor type, IL-8, and IL-6, and directly regulates the function and differentiation of MDSCs within the tumor microenvironment (30, 31). Moreover, MDSCs derived from hypoxic tumor regions show stronger immunosuppressive function than splenic MDSCs. The phenomenon is mostly due to the HIF-1α-driven increased arginase activity and nitric oxide production of tumor MDSCs (32). Several studies have confirmed that hypoxia is strongly associated with the selective accumulation of Tregs in tumors, which not only suppress antitumor response but also promote neo-angiogenesis (33–35). The most direct evidence for hypoxia providing a tolerogenic tumor microenvironment for Tregs is the fact that hypoxia-driven HIF-1α strongly increases the expression of forkhead box P3 (Foxp3), which is a distinct marker and a master regulator in the development and function of Tregs (36–40). Tumor cells under hypoxia can also produce high levels of C-C motif chemokine ligand 28, which is an important chemokine to selectively attract CXCR10-positive Tregs in tumors resulting in antigen tolerance and angiogenesis (34).

Beside regulation of immunosuppressive MDSCs and Tregs within the tumor, recent studies showed that hypoxia promotes immune evasion through HIF-1α-dependent upregulation of immune checkpoint proteins in tumors (26). Of interest, three important checkpoint molecules, such as cluster of differentiation 47 (CD47), programmed death ligand 1 (PD-L1), and human leukocyte antigen G (HLA-G), have been shown by multiple reports to be modulated by hypoxic conditions and to contribute to an immunosuppressive microenvironment in tumors.

### Hypoxia-Driven CD47 Expression in Tumors

Cluster of differentiation 47, also known as integrin-associated protein, has been identified as a membrane protein interacting with β3 integrin, thrombospondin-1, and signal regulatory protein-alpha (SIRPα) to regulate various important cellular functions, including cell migration, cytokine production, T cell activation, and cancer metastasis (41–45). Overexpression of CD47 has been found in a number of tumors, such as acute myeloid leukemia, non-small cell lung cancer, bladder cancer, non-Hodgkin’s lymphoma, and breast cancer (46–53). Based on the analysis of datasets derived from thousands of primary tumors of breast cancer patients, CD47 expression was not only significantly correlated with patient survival but also correlated with the expression of HIF-1α targeting genes (53). A recent study from Semenza’s group showed that HIF-1α activated transcription of CD47 in mesenchymal triple-negative primary breast cancer cells (SUM159) and promoted the breast cancer stem cell phenotype, which further protected cancer cells from phagocytosis by bone marrow-derived macrophages (53). Thus, hypoxic cancer cells evade innate immunity through HIF-1α-dependent expression of CD47. CD47 is emerging as a negative checkpoint for innate immunity and subsequent adaptive immunity in tumors (54). One important mechanism for CD47-mediated immune evasion is that binding of CD47 to SIRPα, which is abundantly expressed on myeloid-linage hematopoietic cells such as TAMs and MDSCs, causes phosphorylation of the SIRPα ultimately resulting in delivering an anti-phagocytic signal (46, 48, 54, 55). The CD47–SIRPα axis not only directly functions as a negative checkpoint of innate immunity but also affects adaptive immunity. In a series of studies, anti-CD47 blockade significantly increased the presence of IFN-γ-expressing antigen-specific CD8^+^ T cells and promoted T cell-mediated destruction of tumor cells (56, 57). Thus, the CD47–SIRPα axis has become an attractive target for developing novel cancer immunotherapies, and anti-CD47 blockades are currently investigated by several clinical trials in various solid tumors (54) (Table 1).

**Table 1:** List of drugs targeting A2A adenosine receptor (A2AR), cluster of differentiation 47 (CD47), and CD73 currently being investigated in clinical trials.

Target	Drug(s)	Details of drug	ClinicalTrials.gov Identifier	Title	First posted date	Disease(s)
A2AR	NIR178 (in combination with PDR001)	NIR178: small molecule adenosine receptor antagonist	NCT03207867	A phase 2 study of NIR178 in combination with PDR001 in patients with solid tumors and non-Hodgkin lymphoma	July 5, 2017	Solid tumors, non-Hodgkin lymphoma
	
		PDR001: anti-PD-1 monoclonal antibody				
	
	PBF-509 (in combination with PDR001)	PBF-509: orally bioavailable A2AR antagonist	NCT02403193	Trial of PBF-509 and PDR001 in patients with advanced non-small cell lung cancer (NSCLC) (AdenONCO)	March 31, 2015	Advanced NSCLC
	
	CPI-444 (in combination with atezolizumab)	CPI-444: orally bioavailable A2AR antagonist	NCT02655822	Phase 1/1b study to evaluate the safety and tolerability of CPI-444 alone and in combination with atezolizumab in advanced cancers	January, 14 2016	NSCLC, malignant melanoma, renal cell cancer triple negative breast cancer, colorectal cancer, bladder cancer, metastatic castration-resistant prostate cancer
	
		Atezolizumab: fully humanized, engineered monoclonal antibody of IgG1 isotype against programmed death ligand 1 (PD-L1)				

CD47	Hu5F9-G4	Hu5F9-G4: monoclonal antibody against CD47	NCT02678338	CAMELLIA: anti-CD47 antibody therapy in hematological malignancies	February 9, 2016	Acute myeloid leukemia, myelodysplastic syndrome
	
	Hu5F9-G4 (in combination with azacitidine)	Hu5F9-G4 Azacitidine: chemical analog of cytidine	NCT03248479	Hu5F9-G4 monotherapy or Hu5F9-G4 in combination with azacitidine in patients with hematological malignancies	August 14, 2017	Acute myeloid leukemia, myelodysplastic syndromes
	
	Hu5F9-G4 (in combination with cetuximab)	Hu5F9-G4 Cetuximab: epidermal growth factor receptor (EGFR) inhibitor	NCT02953782	Trial of Hu5F9-G4 in combination with cetuximab in patients with solid tumors and advanced colorectal cancer	November 3, 2016	Colorectal neoplasms, Solid tumors
	
	Hu5F9-G4	Hu5F9-G4	NCT02216409	Phase 1 trial of Hu5F9-G4, a CD47-targeting antibody	August 15, 2014	Solid tumor
	
	Hu5F9-G4 (in combination with rituximab)	Hu5F9-G4 Rituximab: monoclonal antibody against CD20	NCT02953509	Trial of Hu5F9-G4 in combination with rituximab in relapsed/refractory B-cell non-Hodgkin’s lymphoma	November 2, 2016	Lymphoma, non-Hodgkin lymphoma, large B-cell, diffuse indolent lymphoma
	
	CC-90002	CC-90002: a monoclonal antibody against CD47	NCT02641002	A study of CC-90002 in subjects with acute myeloid leukemia (AML) and high-risk myelodysplastic syndrome (MDS)	December 29, 2015	Leukemia, myeloid, acute myelodysplastic syndromes
	
	CC-90002	CC-90002 and rituximab	NCT02367196	A phase 1, Dose Finding Study of CC-90002 in subjects with advanced solid and hematologic cancers	February 20, 2015	Hematologic neoplasms
	
	TTI-621 (in combination with rituximab or nivolumab)	TTI-621: soluble recombinant antibody-like fusion protein, SIRPa-Fc Rituximab Nivolumab: human IgG4 anti-PD-1 monoclonal antibody	NCT02663518	A trial of TTI-621 for patients with hematologic malignancies and selected solid tumors	January 26, 2016	Hematologic malignancies solid tumor
	
	TTI-621	TTI-621	NCT02890368	Trial of intratumoral injections of TTI-621 in subjects with relapsed and refractory solid tumors and mycosis fungoides	September 7, 2016	Solid tumors, mycosis fungoides, melanoma Merkel cell carcinoma, squamous cell carcinoma, breast carcinoma, human papillomavirus-related malignant neoplasm soft tissue sarcoma
	
	ALX148	ALX148: fusion protein comprised of two high affinity CD47 binding domains of SIRPα linked to an inactive Fc region of human immunoglobulin	NCT03013218	A study of ALX148 in patients with advanced solid tumors and lymphoma	January, 6 2017	Metastatic cancer, solid tumor, advanced cancer, non-Hodgkin lymphoma

CD73	MEDI9447 (in combination with MEDI4736)	MEDI9447: monoclonal antibody against CD73	NCT02503774	MEDI9447 Alone and in combination with MEDI4736 in adult subjects with select advanced solid tumors	July 21, 2015	Solid tumors
	
		MEDI4736: monoclonal antibody against PD-L1				
	
	MEDI9447 (in combination with Durvalumab, Tremelilumab, and MEDI 0562)	MEDI 9447 MEDI 0562: monoclonal antibody binding to OX40 Durvalumab: monoclonal antibody targeting PD-L1 Tremelilumab: monoclonal antibody against CTLA-4	NCT03267589	Trial in patients with relapsed ovarian cancer	August 30, 2017	Ovarian cancer

### Hypoxia-Driven PD-L1 Expression in Tumors

One crucial mechanism by which cancer cells block antitumor immunity is through expression of PD-L1, which binds to the cell surface checkpoint receptor PD-1 on effector T cells to inhibit their activation (58). Accumulated studies confirm that hypoxia can strongly induce HIF-1α-dependent PD-L1 expression on tumor cells, macrophages, and dendritic cells (59–61). In human DU145 metastatic prostate and MDA-MB-231 metastatic breast carcinoma cells, hypoxia-induced expression of PD-L1 has been confirmed to be HIF-1α-dependent (59). The elevated expression of PD-L1 in cancer cells under hypoxic conditions leads to increased apoptosis of cultured cytotoxic T lymphocytes (CTLs) and Jurkat leukemia T cells (59). These observations indicate a mechanism by which hypoxic tumors upregulate PD-L1 expression on tumor cells to promote immune escape from CTLs. A study of B16-F10 melanoma-bearing mice models showed that hypoxia also selectively caused a rapid up-regulation of PD-L1 on splenic MDSCs (60). The up-regulation of PD-L1 in MDSCs under hypoxia was confirmed to be dependent on HIF-1α but not HIF-2α (60). Blockade of PD-L1 under hypoxia enhanced MDSC-mediated T cell activation and was accompanied by decreased production of IL-6 and IL-10 by MDSCs (60). The potential mechanism of hypoxic stress upregulating PD-L1 in tumors may rely on simultaneous binding of HIF-1α and pyruvate kinase M2 (PKM2) to hypoxia response elements (HRE) in the PD-L1 promoter (61). As shown in the study of O’Neill’s group, the inhibition of PKM2 by a small reagent or specific siRNA could lead to downregulation of PD-L1 expression on macrophages, MDSCs, and tumor cells (61). Thus, hypoxia can promote an immunosuppressive microenvironment by recruiting MDSCs to hypoxic regions and increase checkpoint PD-L1 expression on MDSCs and tumor cells. According to the study of a large number of malignant primary tumor tissues from pheochromocytomas and paragangliomas, PD-L2 expression but not PD-L1 expression is significantly associated with stronger hypoxia-driven HIF-1α and carbonic anhydrase 9 (CAIX) (62). These studies suggest that simultaneous blockade of PD-L1/PD-L2 along with inhibition of HIF-1α may represent a promising approach to enhance the activity of cytotoxic T cells.

### Hypoxia-Driven HLA-G Expression in Tumors

The non-classic major histocompatibility complex (MHC) class I molecule is known as an immune checkpoint molecule with specific relevance in cancer immunotherapy (63, 64). HLA-G is a crucial MHC-I molecule and plays an essential role in maintaining immune tolerance and inhibiting the functions of immunocompetent cells to support tumor cells escape from immunosurveillance (64). The immunosuppressive function of HLA-G is mediated by the direct binding of HLA-G to relevant inhibitory receptors. Three HLA-G receptors have been identified: immunoglobulin-like transcript 2 (ILT2, CD85j) expressed by B cells, T cells, natural killer cells (NK cells), and myelomonocytic cells, ILT4 (CD85d) expressed by dendritic cells, monocytes, and macrophages, and KIR2DL4 (CD158d) expressed by NK cells (65, 66). Through these inhibitory receptors, HLA-G can interact with B cells, T cells, NK cells, and antigen-presenting cells, and exert its immunosuppressive functions at different stages of the immune response (65, 66). HLA-G expression is very restricted in adult normal tissues, but is frequently induced in numerous malignant tumors, such as glioblastoma, melanoma, and cervical tumors, contributing to their immune escape (67–70). The expression of HLA-G on cancer cells is also found to associate with a higher tumor grade and poor prognosis, such as in primary and metastatic ovarian tumors and primary colorectal tumors (71–73). Several HREs have been identified in HLA-G promoter and non-promoter regions (74). Recent studies showed that the expressions of HLA-G mRNA and protein were upregulated in HLA-G-negative cancer cells *via* HIF-1α under hypoxic conditions (74–76). However, in cancer cells expressing HLA-G constitutively, hypoxia decreases HLA-G gene expression (73). It is still unclear why the expression patterns of HLA-G in HLA-G-negative and HLA-G-positive cancer cells are so different upon hypoxic stress. Since HLA-G is considered an immune checkpoint molecule, augmentation of HLA-G expression in hypoxic tumor cells may contribute to immunosuppression in tumors. Up till now, several reports showed that hypoxia upregulates HLA-G expression in human cancer cells, but very few studies have been published on the effects of hypoxia on other MHC-I molecules in tumors. A recent study showed that combining hypoxic stress and glucose deprivation increased surface expression of HLA-E in human and Qa-1 in mouse tumor cells (77). Further studies are needed to address how hypoxic microenvironment modulates MHC-I and MHC-II molecules in tumors.

## Hypoxia-Driven Immunosuppressive Metabolites

In order to support rapid growth of tumor cells, hypoxic signaling permits tumor cells to sense and adapt to low O_2_ stress and carbon source availability by re-programming their metabolism and gene expression *via* HIF’s transcriptional regulation. Under hypoxia, tumor cells switch to glycolysis to continue ATP production and prevent O_2_-dependent oxidative phosphorylation (78). Also, metabolic intermediates from glycolysis can be utilized for the biosynthesis of other macromolecules. HIF-1α plays a critical role in the glycolytic switch to increase glucose utilization in hypoxic tumor cells by upregulating the expression of glucose transporters and glycolytic enzymes, such as glucose transporters 1 and 3 (GLUT1 and GLUT3), pyruvate dehydrogenase, lactate dehydrogenase A (LDHA), phosphoglycerate kinase 1, and hexokinases 1 (HK1) (78). The increase of these gene’s expressions alters glucose metabolism and prevents glucose entry into the tricarboxylic acid (TCA) cycle and reducing acetyl coenzyme A (CoA) production from pyruvate (78). Reprogramming glucose flux is considered as a major factor to shape the tumor microenvironment *via* increase of HIF-1α levels in rapidly growing tumor cells within hypoxic regions.

### Hypoxia-Driven Acid–Base Regulation and Production of Immuno-Modulatory Lactate

A notable feature of solid tumors is the presence of an acidic extracellular tumor microenvironment mainly due to the production of large amounts of acidic metabolites by glycolytic tumor cells (79). As a consequence of glycolysis, hypoxic tumor cells upregulate LDHA and convert pyruvate into lactic acid, which results in increased tumor acidosis (79–81). At the same time, tumor cells also adapt to intracellular acidification by enhancing export of lactate/H^+^. MCT1 and MCT4 are major players from the monocarboxylate transporter (MCT) family to preferentially transport lactate/H^+^ across the plasma membrane (82, 83). Noteworthy, MCTs are among those genes that are upregulated under hypoxic condition (82–84). The export of lactate/H^+^ by MCT has been found to not only contribute to the acidosis of tumor microenvironment but also to promote tumor cell metastasis, angiogenesis, and suppressing immunosurveillance (82, 83).

**Figure 2:**
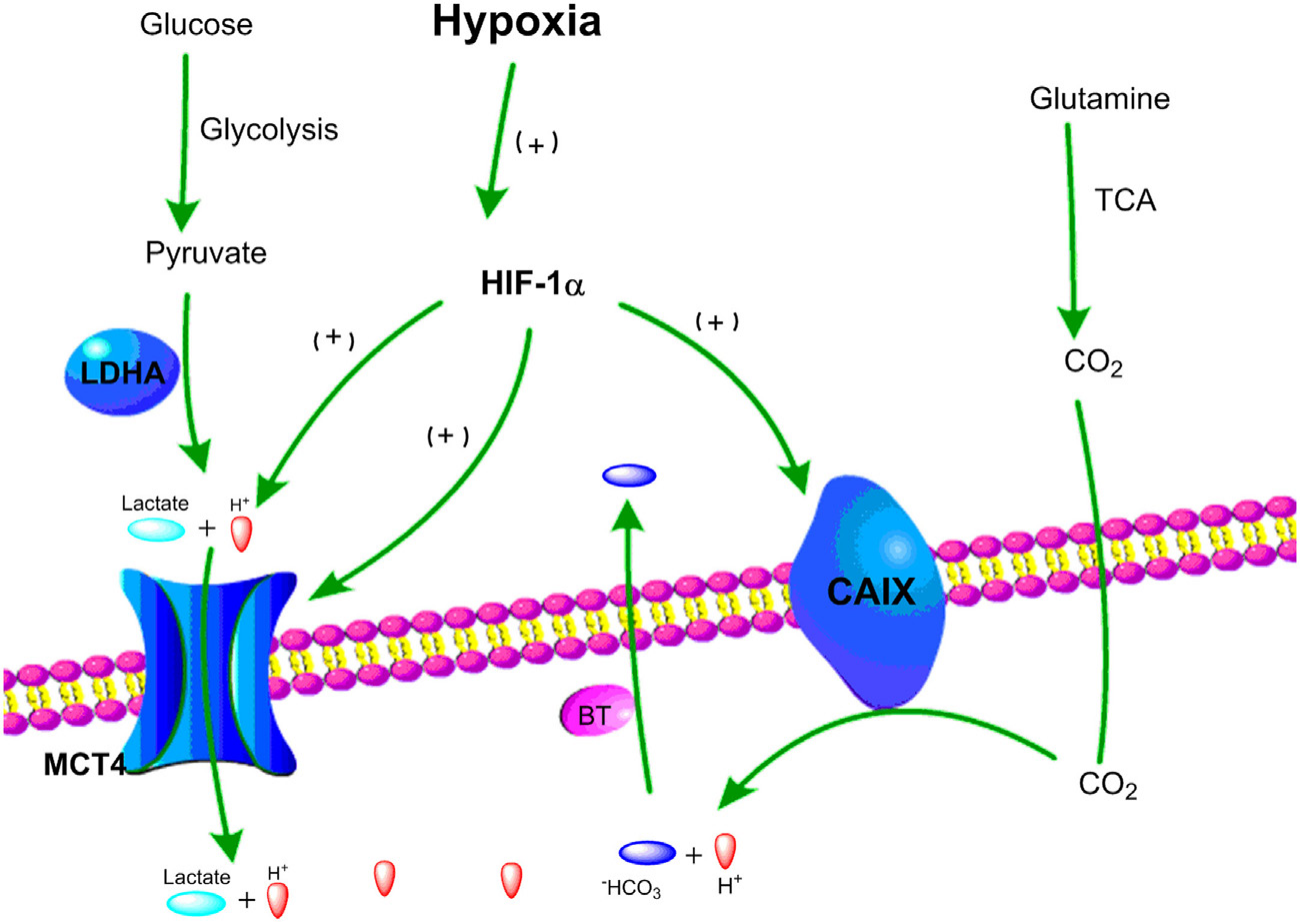
Schematic representation of two major pathways of hypoxia-driven extracellular acidity by tumor cells. (1) Under hypoxic conditions, glucose goes through glycolysis and produces pyruvate, which is mainly converted to lactic acid by lactate dehydrogenase A (LDHA). Lactic acid is exported by MCT4 resulting in acidification of the tumor microenvironment. (2) Under hypoxic conditions, glutamine goes through tricarboxylic acid (TCA) cycle and releases CO_2_, which can be converted into HCO_3_^−^ and H^+^. HCO_3_^−^ is transported back to tumor cells by bicarbonate transporters (BT), and accumulation of extracellular H^+^ leads to tumor acidosis.

In order to provide sufficient energy for rapidly growing tumors, cancer cells use substitute carbon sources, like glutamine, to promote the TCA cycle and maintain oxidative phosphorylation under hypoxic microenvironment (85, 86). Besides lactate, increased levels of CO_2_ generated by oxidative metabolism are another major source for tumor acidity. Indeed, CO_2_ produced by the TCA cycle and the pentose phosphate pathway under hypoxia can be hydrated by carbonic anhydrases (CA) and converted into bicarbonate (HCO3^−^) and protons (H^+^) (87, 88). To balance intracellular acidosis in hypoxic tumor cells, the HCO3^–^ are imported back into cells through bicarbonate transporters and anion exchange, but the H^+^ remains extracellularly and contributes to an increasingly acidic tumor microenvironment (88, 89). Carbonic anhydrase IX (CAIX) plays a key role in pH regulation in hypoxic cancer cells as its expression is induced by hypoxia *via* HIF-1α (89, 90). Together, the key pH regulatory components, such as MCT4 and CAIX, are upregulated in hypoxic tumor cells leading to the acidification of the tumor microenvironment (Figure 2).

Due to the limitation of technology to directly and accurately measure the pH within the hypoxic region of tumors, our current knowledge on the effects of cancer acidity on T cells is based on studies applying *in vitro* cultures to stimulate immune cells at low pH. Under low pH conditions (≤6.6), the secretion of IL-2, tumor necrosis factors, and IFN-γ was impaired in T lymphocytes upon stimulation with anti-CD3 antibody and phytohemagglutinin (PHA) (79, 91, 92). However, IFN-γ R2 chain (IFNR2) and CTLA-4 expressions were upregulated under the same condition which rendered tumor-infiltrating T cells sensitive to negative regulatory signaling. Moreover, the cytotoxicity of antigen-specific T cells appeared to be highly sensitive to low pH. In the *in vitro* study by Takahashi’s group, the cytotoxic activity of CD8+ CTLs decreased in a pH-dependent manner and the induction of functional CTLs were markedly inhibited under low pH (93). Tumor acidity also plays a critical role in assisting tumor cells escape from NK cell-mediated cytolysis. Several *in vitro* studies showed that the cytotoxic activities of NK cells and lymphokine-activated killer (LAK) cells were markedly reduced under acidic conditions (79, 94–96). Indeed, NK activation and LAK generation by IL-2 were inhibited at an extracellular culture condition below pH 7.2 (97). Notably, the low environmental pH caused irreversible damage to NK and LAK cells resulting in a permanent decrease of the cytotoxic activity of these cells, which could not be recovered upon switching to higher pH culture medium (97). A handful studies showed that tumor acidity contributed to the direct induction of a series of pro-inflammatory molecules in tumor-associated myeloid cells (79, 98). Extracellular acidosis has been shown to activate PI3K/Akt and ERK pathways and led to the stimulation of human neutrophils, which resembled the functional profile of MDSCs (99).

Although lactic acid is not the major contributor to the extracellular acidification of tumors, more and more data underline the important role of lactate as a “signaling molecule” involved in regulating cancer cell survival, proliferation, and metastasis (100, 101). Importantly, current studies indicate that lactate is emerging as an important immunosuppressive metabolite promoting escape of immune surveillance in hypoxic tumors (101). Lactate generated by hypoxic tumor cells was found to strongly inhibit the anti-tumor immune response *via* attenuating the cytotoxic activity of human CTLs (102, 103) and NK cells (104, 105). Previous studies also showed that lactate not only inhibited dendritic cells releasing cytokines but also impeded the differentiation and activation of monocyte-derived dendritic cells (106–109). A study using a pancreatic cancer mouse model further demonstrated that tumor-derived lactate could directly inhibit cytolytic function of NK cells. Moreover, lactate could recruit and increase the number of MDSCs in tumor to indirectly inhibit NK cytotoxicity (104). Lactate also acts as an important signaling molecule to promote the production of cytokines such as IL-23 and IL-6 contributing to tumor-associated inflammation (104, 109). Recently, lactate has been identified as a valuable prognostic marker of disease progression and poor patient survival especially in primary carcinomas, including cervical cancer, rectal adenocarcinoma, glioblastoma, and prostate cancer (110–117).

### Hypoxia-Driven Production of Immunosuppressive Adenosine

**Figure 3:**
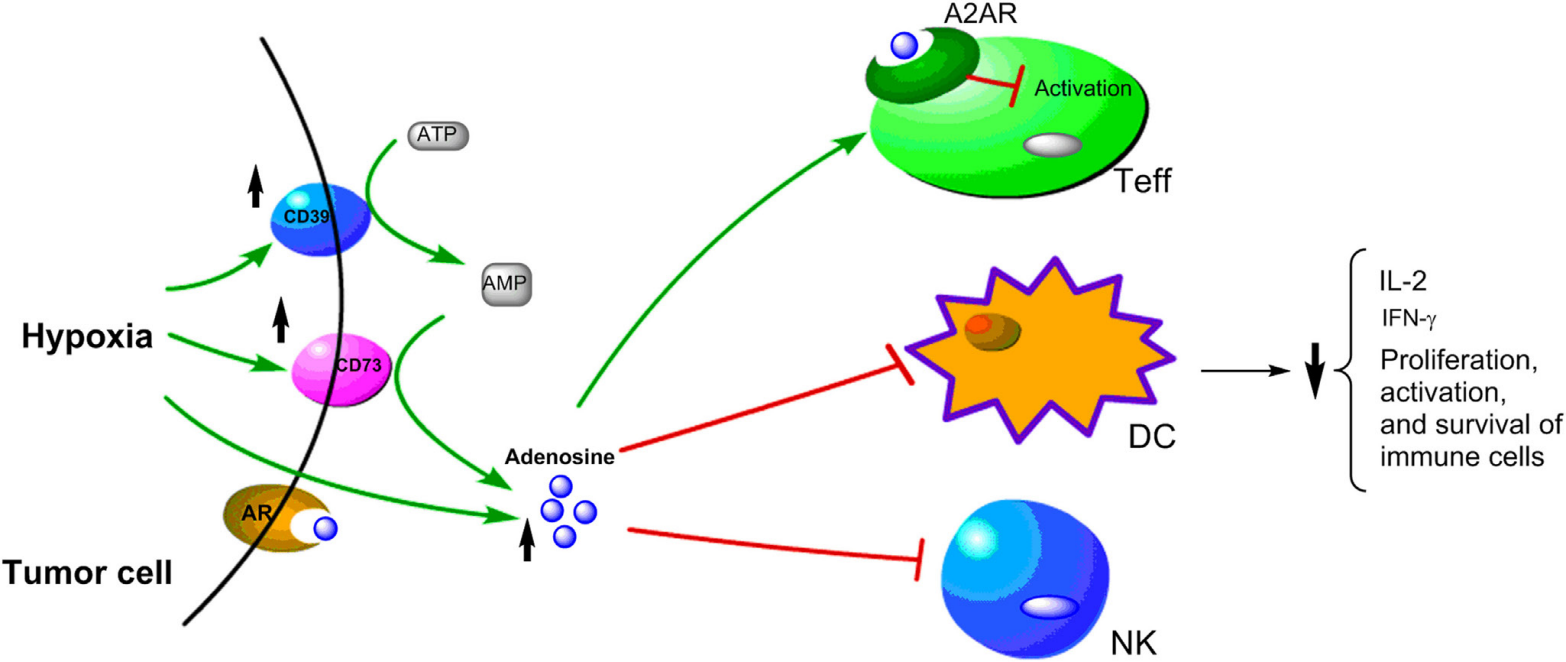
Schematic representation of hypoxia upregulating immunosuppressive adenosine signaling pathway in cancer cells. Under hypoxic conditions, the upregulation of CD39 and CD79 expressions lead to increase of adenosine, which has been shown to have immunosuppressive effects on Teff, DC, and NK cells.

One important immunomodulatory metabolite which accumulates in hypoxic tumors is adenosine. It is known that hypoxia can upregulate the expression of CD39 and CD73. Under hypoxia, the nucleotide metabolism mainly undergoes phosphohydrolysis *via* ectonucleoside triphosphate diphosphohydrolase CD39 that converts ATP/ADP to AMP. Then, 5′-ectonucleotidase CD73 converts AMP to adenosine (118–120). The CD39–CD73-adenosine signaling represents an important pathway to generate extracellular adenosine. Moreover, HIF-1α inhibits the intracellular adenosine kinase to prevent re-phosphorylating adenosine to AMP resulting in elevated levels of intracellular adenosine. The high level intracellular adenosine is subsequently transported into extracellular space (120, 121). Hypoxia also enhances adenosine signaling by increasing the expression of A2A adenosine receptor (A2AR), which is a key G-protein coupled receptors conducting adenosine singling (121, 122). The adenosine accumulated in the tumor microenvironment acts as a negative regulator for both the activation and effector phases of the anti-tumor T cell response (123–126). The binding of adenosine to A2AR on T cells can lead to T cells apoptosis, which contributes to tumor immune evasion (125–127). Thus, the adenosine–CD73 axis represents a hypoxia-driven immunosuppressive mechanism in solid tumors (Figure 3). The high concentration of extracellular adenosine is usually present in cancer tissues, which is an important mediator in the alteration of immune cell functions to drive the immunosuppressive microenvironment in tumors (125, 128).

## Targeting Hypoxia-Driven Metabolic Pathways to Enhance the Efficacy of Immunotherapy of Tumors

As a hallmark of solid tumors, hypoxia is known to mediate aggressive, metastatic, and resistant characteristics. For a long time, hypoxia-relevant signaling pathways have been among the most attractive therapeutic targets in cancer drug development. Various approaches have been proposed to target hypoxic tumor cells, including hypoxia-activate prodrugs, gene therapy, recombinant anaerobic bacteria, small inhibitors specifically targeting HIFs, or targeting important downstream components of hypoxic pathways such as, mTOR and URP pathways (129–131). These approaches have been comprehensively discussed in multiple reviews (129–131). Although much effort has been put into investigating drugs targeting hypoxic pathways in clinical trials; results have been generally disappointing (132, 133). There is an urgent need to improve our understanding of the complexity of hypoxic pathways and their roles in solid tumors for drug development.

To date, there are no approved drugs that directly inhibit the HIF pathway. It is known that hypoxia-driven HIF-1 regulates a highly complex network involving multiple signaling cascades and overlapping mechanisms. The failure of clinical studies targeting hypoxic pathways in tumors may be partially due to the lack of specificity of inhibitors and redundancy in hypoxic signaling/metabolism, which impede the efficacy of drugs. Most of the reported HIF-1 inhibitors were originally designed to target other molecules, and they were found to have HIF-1 inhibitory effect later. The development of specific inhibitors of HIF-1 represents a challenge, which is mainly due to the difficulty of targeting transcription factors to selectively interrupt protein–DNA or protein–protein interactions without affecting other pathways. Although it is assumed that hypoxic regions exist in most solid tumors, the inhibitors of HIF-1 or other hypoxia-relevant molecules may be less effective in the patients that do not have high levels of HIF-1. Therefore, the lack of specific patient selection may also contribute to the failure of those clinical trials of HIF-1 inhibitors, in which the selection of patients were not directly based on the HIF levels in tumors (129–133). The hypoxic microenvironment is considered to be a major contributor but not a driving force for tumor progression and metastasis. Thus, understanding the mechanism of hypoxic pathways and their interaction with other pathways in tumors is of particular importance. Future directions would be directed toward developing potent and more specific inhibitors targeting hypoxia-relevant molecules, which can be used in combination therapies and will hopefully overcome hypoxia-driven resistance.

Currently, one of the most promising treatments for metastatic melanoma and several other cancers is checkpoint blockade immunotherapy. In contrast to the direct cytotoxic effects of chemotherapy, checkpoint blockade relies on antigen-specific T cell responses by blunting tumor-induced immunoregulatory mechanisms. This form of treatment has provided durable, long-lasting responses in many patients, largely due to the persistence and adaptability of the immune system. As summarized in previous sections, hypoxia plays a crucial role in immunoregulatory networks to promote an immunosuppressive tumor microenvironment. Interventions in several critical hypoxic axes emerge as promising adjuvants for a variety of immunotherapies.

One crucial enzyme mediating hypoxia-driven immunosuppression in solid tumor is CAIX. CAIX is upregulated by HIF-1α and represents a prototypic tumor-associated antigen. Overexpression of CAIX was found in metastatic renal cell carcinoma (RCC), and decreased CAIX levels are independently associated with poor survival in advanced RCC (134). A monoclonal antibody specifically targeting CAIX has been developed. In the study from Marasco’s group, human anti-CAIX mAbs not only inhibited CAIX enzymatic activity but also promoted immune-mediated killing of RCC by NK cell-mediated antibody-dependent cell-mediated cytotoxicity, complement-dependent cytotoxicity, and macrophage-mediated antibody-dependent cell-mediated cytotoxicity. This study demonstrated that targeting CAIX could induce an immune response to inhibit CAIX-positive tumor growth *in vivo* through tumor infiltration of NK cells and activation of T cells (135). Thus, the anti-CAIX reagent presents a therapeutic potential for the unmet medical need of targeted killing of HIF-1α-driven CAIX-positive RCC. Girentuximab has been developed as a chimeric monoclonal antibody drug specifically against CAIX. The current report from the phase 3 clinical trial of girentuximab in clear cell renal cell carcinoma (ccRCC) showed that participants treated with girentuximab had no statistically significant disease-free survival (hazard ratio, 0.97; 95% CI, 0.79–1.18) or OS advantage (hazard ratio, 0.99; 95% CI, 0.74–1.32) compared to the placebo group (136). Although girentuximab had no clinical benefit as adjuvant treatment for patients with high-risk ccRCC, it is still not clear whether girentuximab facilitates releasing the immunosuppression driven by hypoxic CAIX in tumors.

Although immune checkpoint blockade, such as anti-PD-1 therapy, has led to dramatic responses in some cancer patients, overall response rates are still less than 30% (58), which likely reflects the fact there are multiple layers and redundant mechanisms of immune evasion in solid tumors. Thus a single targeted therapy or immunotherapy is insufficient to restore antitumor immunity to clear various highly heterogeneous tumor cells. The hypoxia-dependent metabolic reprogramming also contributes to immune evasion, as T cells subjected to glucose deprivation (due to increased glucose uptake by hypoxic cancer cells) have diminished antitumor effector functions (26).

The binding of hypoxia-driven adenosine to A2AR could protect tumor cells from immune clearance by inhibiting T cells response. High level of A2AR expression has been confirmed in primary tumor tissues of head and neck squamous cell carcinoma (HNSCC), and it was significantly correlated with HIF-1α, CD73, CD8, and Foxp3 (137). Moreover, increased expression of A2AR on tumor infiltrating immune cells has been shown to correlate with advanced pathological grade, larger tumor size and positive lymph node status in primary HNSCC (137). The *in vivo* study of HNSCC mouse model showed that the A2AR antagonist, SCH58261, not only delayed the tumor growth but also significantly reduced the population of CD4+ Foxp3+ Tregs and increased the anti-tumor response of CD8+ T cells in HNSCC tumors (137). This preclinical study indicates that A2AR blockade can be a potential strategy to enhance immunotherapy in HNSCC. Other key mediators in hypoxia-driven immunosuppression that also draw a great attention for drug development include CD73 and CD47. Multiple therapeutic approaches targeting A2AR, CD73, or CD47 are currently being investigated in clinical trials (Table 1). Given the unique roles of these targets in modulating immunosuppression within hypoxic tumors, the specific therapeutics targeting these are expected to work as potential boosters to synergize with other immunotherapies and offer opportunities to enhance anti-tumor activity of immune effector cells.

## Author Contributions

YQ, SP, JR, and YL wrote the review. All authors approved the final version of the manuscript.

## Conflict of Interest Statement

The authors declare that the research was conducted in the absence of any commercial or financial relationships that could be construed as a potential conflict of interest.
